# Effects of Rice–Frog Co-Cropping on the Soil Microbial Community Structure in Reclaimed Paddy Fields

**DOI:** 10.3390/biology13060396

**Published:** 2024-05-30

**Authors:** Yunshuang Ma, Anran Yu, Liangliang Zhang, Rongquan Zheng

**Affiliations:** 1 Provincial Key Laboratory of Wildlife Biotechnology and Conservation and Utilization, Zhejiang Normal University, Jinhua 321004, China; mys000408@zjnu.edu.cn (Y.M.); yuanran@zjnu.edu.cn (A.Y.); 2 Xingzhi College, Zhejiang Normal University, Jinhua 321004, China; liangzhang588@gmail.com

**Keywords:** rice–frog co-cropping, reclaimed land, soil fertility, microbial community structure

## Abstract

**Simple Summary:**

The utilization of reclaimed land and enhancement of its productivity are crucial for alleviating China’s food production shortage. Previous research has indicated that co-cultivating rice with frogs can enhance soil fertility. However, there is limited research on the use of integrated rice–frog farming techniques to improve soil fertility on reclaimed barren land for enhanced food production efficiency. Therefore, this study evaluated the impact of rice–frog co-cultivation on soil fertility and microbial diversity of reclaimed land. Experimental plots were established for rice monoculture, low-density rice–frog co-cultivation, and high-density rice–frog co-cultivation. The study found that during the rice maturity period, the soil fertility in the plots with high-density rice–frog co-cultivation was significantly higher compared to the rice monoculture plots. Additionally, rice–frog co-cultivation increased the soil microbial diversity and altered the structure of the microbial community compared to the rice monoculture. This research indicates that compared with rice monoculture, rice–frog co-cultivation can enhance the soil fertility and microbial diversity of reclaimed land. This study provides a theoretical basis for the construction of integrated farming models on reclaimed land, offering new perspectives and opportunities for the efficient utilization of such land.

**Abstract:**

Utilizing and improving the productivity of reclaimed land are highly significant for alleviating the problem of food production shortage in China, and the integrated rice–frog farming model can improve soil fertility. However, there are few studies on the use of integrated rice–frog farming technology to improve the fertility of reclaimed land and increase its efficiency in food production. Therefore, this study was conducted to evaluate the effects of the rice–frog co-cropping mode on the soil fertility and microbial diversity of reclaimed land. A rice monoculture group (SF), low-density rice–frog co-cropping group (SD, 5000 frogs/mu, corresponds to 8 frogs/m^2^), and high-density rice–frog co-cropping group (SG, 10,000 frogs/mu, corresponds to 15 frogs/m^2^) were established and tested. The contents of total nitrogen, soil organic matter, available potassium, and available phosphorus of the soil in the SG group were significantly higher than those in the SF group (*p* < 0.05) in the mature stage of rice. Compared with the SF group, the SD and SG groups improved the soil microbial diversity and changed the structure of the microbial community. This study indicates that compared with the rice monoculture mode, the rice–frog co-cropping pattern can improve the soil fertility, as well as microbial diversity, of reclaimed land.

## 1. Introduction

Rice (*Oryza sativa* L.), as a major food crop, occupies an important position in the Chinese economy. With the growth in the global population, the need for food is becoming an increasingly pressing issue [1,2], and efforts to secure food production are highly significant. The reclamation and utilization of idle land have a positive impact on agricultural development and other aspects [3]. However, most reclaimed land is relatively infertile and has poor biological conditions; this increases the difficulty of crops in adapting to it and results in much lower productivity than normal arable land [4,5,6,7]. Rapidly improving the soil fertility of reclaimed croplands and optimizing and reconstructing their soil chemical properties and biological conditions have become key issues of ecological research on this cropland.

The cultivation of species together is a way to intensify agriculture. Rice–aquatic animal farming, a type of species co-cultivation, is widely practiced and researched [8]. Currently, there is a growing trend in the global cultivation of both irrigated and paddy fields, accompanied by the adoption of co-cropping models involving rice and aquatic animals. While extensive research has been conducted on rice–fish [9,10], rice–turtle [11], and rice–crab co-cropping [11], limited studies have focused on amphibian–rice co-cropping [12]. The rice–frog co-cropping model, which involves the co-cultivation of rice and frogs, can simultaneously produce green rice and high-quality animal products; it is considered an effective way to develop ecological agriculture and increase the diversity of agricultural models [13,14,15]. The rice–frog co-cropping model can effectively counteract losses in nitrogen (N), phosphorus (P), and other elements in paddy fields, thus improving the fertility of the soil [16]. Additionally, compared with traditional agriculture, the rice–frog co-cropping model can maintain stable contents of soil organic matter (SOM), total nitrogen (TN), and other nutrients with reduced inputs of fertilizer [17,18].

Microorganisms are the primary participants in the material flow and energy cycles in paddy ecosystems [19,20] and are primarily involved in regulating the conversion of nutrients, such as N, P, and potassium (K), which are essential for the growth of rice, as well as the decomposition and accumulation of SOM [21,22,23]. Therefore, they can be used as key indicators to evaluate the quality of soil and ecosystem functions [24,25,26]. Microorganisms are sensitive to the environment, and anthropogenic activities, crop rotation methods, and planting patterns can affect the composition of their community [27,28,29]. Therefore, soil microbial communities can be used as indicators to track changes in various land management practices, such as those in restoration outcomes [30,31] or evaluations of agricultural management practices [32]. It has been found with different aquaculture species that an integrated rice-farming model can increase the populations of microorganisms in the soil, as well as the diversity of their communities, and improve the availability of nutrients in the soil [13,31,33,34,35,36,37].

The rice–frog cropping pattern is increasingly advocated for owing to its significant economic and ecological benefits [8]. The presence of frogs in paddy fields can influence the microbial activity and community structure of microorganisms in the soil [34]. Despite this, research on how the rice–frog cropping pattern affects soil bacterial communities is still limited. This is of particular concern for the different densities of this cropping pattern for the ecological restoration of reclaimed fields. Therefore, this study aimed to assess the soil chemical properties, as well as the soil bacterial community structure and diversity, in a 0–20 cm soil layer of reclaimed paddy fields at various growth stages of rice under different densities of rice–frog cropping and conventional rice cropping modes. The objectives were to elucidate the impact of the rice–frog cropping pattern on the chemical properties and bacterial community structure of the soil, offer insight into the practical application of this cropping mode, and contribute to the ecological restoration and management of reclaimed paddy fields in the future.

## 2. Materials and Methods

### 2.1. Overview of the Study Area

The experimental site was located in Shafanxiang (119°29’36” E, 28°52’42” N), Jinhua City, Zhejiang Province, China, with an average annual rainfall of 1309 mm and an average of 1810.3 h of annual sunlight, which have uniform durations. This trial site is a reclaimed field of idle land, and the planting management system was one season of late rice per year.

### 2.2. Experimental Design

A previous study [14] on rice–frog co-cropping confirmed that the frog density required to achieve the optimal eco-economic benefits was 60,000 frogs/ha (6 frogs/m^2^). Since the weight of the tiger frog (*Rana rugulosa* Wiegmann, 1834) used in Fang et al. [14]’s study (15 g on average) is much higher than the weight of the dark-spotted frog (*Pelophylax nigromaculatus* Hallowell, 1861) used in this experiment (1–5 g), the density of the frogs was increased in this experiment. Three experimental production fields were selected and divided into a rice monoculture group (SF, 0 frogs/mu), a low-density rice–frog co-cropping group (SD, 5000 frogs/mu, corresponded to 8 frogs/m^2^), and a high-density rice–frog co-cropping group (SG, 10,000 frogs/mu, corresponded to 15 frogs/m^2^) according to the densities of *Pelophylax nigromaculatus*. The variety of rice in the study was “Yongyou No. 31”. Healthy, disease-free frogs that weighed 1–5 g were placed on rice that had been transplanted for 15 days, in sunny weather, after the seedlings had become green and viable. Jindadi feed (Zhejiang Jindadi Bio-technology Co., Ltd., Shaoxing, China) was used as the feed for the frogs in this study. The feeding amount was approximately 5% of the frog’s weight. The frog feed contained ≥40% crude protein, ≥4.5% crude fat, ≤8% crude fiber, ≤18% crude ash, ≥1% total phosphorus, and ≥1.8% lysine. After being put in the paddy field, the frogs were fed at 17:00 every day. No fertilizers or pesticides were applied to all three fields throughout the experimental period. Wooden piles were driven around the experimental field, and polyethylene mesh was used as fences, which were built on the ridge. The fences were 1.0–1.5 m high and buried about 10 cm underground. Two water inlets and outlets were excavated in each experimental paddy field, and the frog fence was made with wire mesh to prevent frogs from escaping. In addition, all experimental plots had a built Skynet to prevent birds from preying on frogs. Therefore, the frogs were neither preyed upon by natural predators nor did they escape from one plot to another, thus avoiding the hazards associated with invasive species. The frogs were soaked in a mild disinfectant (2–3% saline solution) for 5–10 min prior to release [38], and the water quality was maintained as clean and circulated during the breeding process to prevent infection from pathogenic bacteria that could potentially lead to the death of *Pelophylax nigromaculatus* [39]. The experimental period was from July to October.

### 2.3. Sample Collection

The soil samples were first collected on 6 July 2022 (before reclamation), and the soil was also sampled during the four periods of rice tillering, heading, full heading, and maturity stage, with three replicates in each group and a total of nine samples for each period. Each treatment field was sampled using an S-shaped, 5-point sampling method of 0–20 cm of the rice’s inter-root soil, which was mixed as a single sample. All samples were placed on ice to keep them below 0 ℃ and immediately transported back to the laboratory. The samples were numbered as follows: rice monoculture field: SF_1, SF_2, SF_3, and SF_4, which refer to the four stages of tillering, heading, full heading, and maturity of rice, respectively; low-density rice–frog co-cropping field: SD_1, SD_2, SD_3, and SD_4; and high-density rice–frog co-cropping field: SG_1, SG_2, SG_3, and SG_4. After impurities were removed, 5–10 g of each sample was used in the experiments, preserved in 100 mL plastic centrifuge tubes, and stored at −80 °C.

### 2.4. Methods of Measurement

#### 2.4.1. Determination of Soil Chemical Properties

The soil samples of before land reclamation and during the mature period of rice were used to determine the chemical properties. The SOM was determined using the potassium dichromate (K_2_Cr_2_O_7_) method, and the TN was determined using the Kjeldahl method. The available nitrogen (AN), available phosphorus (AP), and available potassium (AK) of the soil were determined by the alkaline hydrolysis diffusion, molybdenum antimony colorimetry, and flame photometry methods, respectively [40].

#### 2.4.2. Extraction of the Total DNA from the Soil

The nucleic acids were extracted using a TGuide S96 Magnetic Bead Kit for Soil/Fecal Genomic DNA Extraction (DP812; Tiangen Biochemistry, Beijing, China), which is suitable for most microbial samples. The concentration of the nucleic acids extracted was measured using an enzyme labeling instrument (Bio Tek, FLX800T, Beijing, China) and amplified according to the assay. The integrity of the PCR products was checked using 1.8% agarose electrophoresis.

#### 2.4.3. Construction of the Library

The PCR amplification, which targeted the V3–V4 variable region of the 16S rRNA gene, was performed using the universal primers 338F (5’-ACTCCTACGGGGAGGCAGCA-3’) and 806R (5’-GGACTACHVGGGGTWTCTAAT-3’) [41]. The PCR was performed with a 10 μL reaction system under the following conditions: 98 ℃ for 30 s of denaturation, 27 cycles at 98 ℃ for 15 s, 50 ℃ for 30 s, 72 ℃ for 5 min of annealing, and 72 °C for 5 min for the final elongation. The PCR products were purified and subjected to *trans*-agarose electrophoresis for positive detection. The libraries were quality tested by the Qsep-400 method. The constructed libraries were sequenced using an Illumina NovaSeq 6000 platform (Illumina, San Diego, CA, USA).

### 2.5. Sequencing Analysis and Microbial Taxonomic Identification

The microbiome was analyzed using QIIME2 v. 2021. 8 [42]. The raw sequence data were decoded using the demux plugin, and the primers were excised using the cutadapt plugin [43]. The sequences were processed by quality filtering, denoising, splicing, and chimera removal using the DADA2 plugin [44]. The sequences were then merged at a 100% sequence similarity to generate characteristic sequence amplicon sequence variants (ASVs) and abundance data tables. The Greengenes database was used to compare the ASV feature sequences with the reference sequences in the database to obtain the taxonomic information that corresponds to each ASV. The ASVs with abundance values below 0.001% of the total number of sequenced samples were removed, and the abundance matrices of the removed rare ASVs were subsequently analyzed. Moreover, the identification results for each sample at each taxonomic level were plotted as bar charts using R Core Team (2018) to visually compare the differences in the ASV numbers and identify the taxonomic statuses of the different samples.

### 2.6. Statistical Analysis

SPSS 21.0 (IBM, Inc., Armonk, NY, USA) and R were used for the statistical analyses. A one-way analysis of variance (ANOVA) was used to compare the soil chemical properties among the groups. A permutational MANOVA (Adonis/PERMANOVA) was used to evaluate the significance of the differences in microbial community structure among the groups. The following seven diversity indices, including the Chao1, Observed_otus, Shannon, Simpson, Pielou_e, and good_coverage indices, were calculated for each sample using QIIME2, and box plots were drawn to compare the abundance and homogeneity of the ASVs among the different samples using Origin Pro (OriginLab, Northampton, MA, USA). Differences in the alpha-diversity indices of the samples from different experimental groups were tested using a *t*-test. A beta-diversity analysis was performed using the UniFrac distance metric to investigate the variation in microbial community structure among the samples. R and QIIME2 were used for this analysis, and differences among the samples were observed with a principal coordinates analysis (PCoA) and clustering analysis (UPGMA). Differences in the relative abundances of the microbial taxa among the groups were detected using the linear discriminant analysis (LDA) effect size (LEfSe). Taxa with significant *p*-values (*p* < 0.05) and LDA scores ≥ 3 were considered to be differentially abundant taxa. BugBase, from Knight lab (Minneapolis, Minnesota, USA), was used to predict the phenotypes of the microbiome samples. The effect of the soil’s chemical properties on the soil’s bacterial community composition was obtained using a redundancy analysis (RDA) and Pearson correlation coefficient analysis. The Tukey test was used to compare the averages of each group. The data are shown as the mean ± SE.

## 3. Results

### 3.1. Properties of the Soils under Three Farming Modes

**Figure 1:**
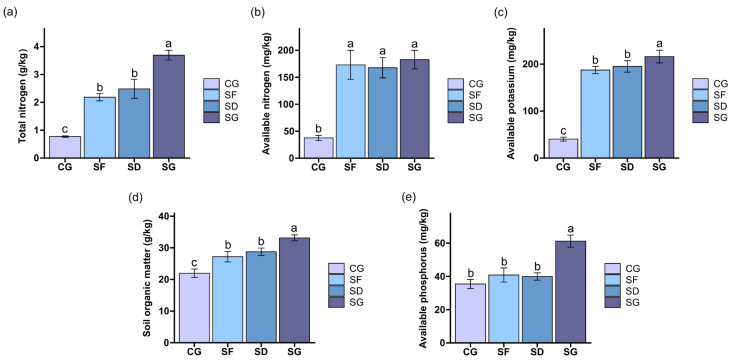
Effects at maturity of the different farming modes on the chemical properties of the soil: (**a**) total nitrogen (TN); (**b**) available nitrogen (AN); (**c**) available potassium (AK); (**d**) soil organic matter (SOM); (**e**) available phosphorus (AP). CG: control group (before reclamation); SF: rice monoculture mode; SD: low-density rice–frog co-cropping mode; SG: high-density rice–frog co-cropping mode. Different lowercase letters within the same period indicate significant differences at the *p* < 0.05 level (Duncan’s test).

The soil before reclamation was used as a control (CG) to observe the effects of different farming modes on the chemical properties of the soil. As shown in Figure 1, the contents of TN, AN, AK, and SOM in the soils for all three farming modes during the period of rice maturity increased significantly compared with those before land reclamation (*p* < 0.05). In the rice maturity period, the contents of TN, AK, SOM, and AP in the soil of the SG group were significantly higher than those in the SD and SF groups (*p* < 0.05), and there was no significant difference between the SD and SF groups. There was no significant difference in the content of AN in the soil among the three groups in the mature stage of the rice.

### 3.2. Bacterial Community Composition of the Soils

**Figure 2:**
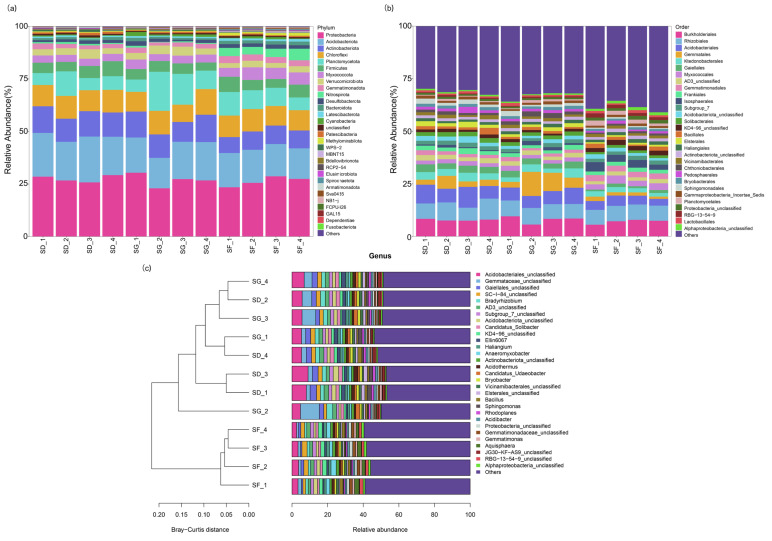
Effects of three different farming modes on the relative abundances of different levels of microbial populations in soil: (**a**) relative abundance of bacteria at the phylum level; (**b**) relative abundance of bacteria at the order level; (**c**) Bray–Curtis distance clustering tree plot of bacteria at the genus level.

A total of 36 samples from 12 groups of soil were sequenced to obtain 3,034,015 pairs of reads. Double-end splicing, quality control, and chimera filtration yielded a total of 2,502,467 clean reads. Each sample yielded a minimum of 62,360 reads and an average of 69,512. The exponential sparsity curve of the operational taxonomic units (OTUs) for the soil bacteria leveled off after a certain depth of sequencing and no longer increased with the sequencing depth, thus indicating that the OTUs detected were sufficiently representative of the microbial community in each sample (Figure S1). A total of 68 bacterial phyla were detected, and the composition and relative abundance of the bacterial community were analyzed at the phylum classification level, as shown in Figure 2a. The primary dominant bacterial phyla in the soil samples of the SF, SD, and SG groups were Proteobacteria and Acidobacteria, which composed 22.97–30.39% and 14.46–21.78% of the total bacteria, respectively. The relative abundance of Proteobacteria in the SD and SG groups was significantly higher than that in the SF group (*p* < 0.01), and the relative abundance of Acidobacteria was the highest in the SD group. In addition, the dominant phyla in the soil samples of the SD, SG, and SF groups also included Actinobacteria, Planctomycetota, and Chloroflexi, with relative abundances of 11.57–13.06%, 11.68–18.57%, and 9.29–10.66%, respectively.

The bacterial community composition and relative abundance were analyzed at the level of order classification, and 445 bacterial orders were detected, as shown in Figure 2b. The primary dominant bacterial orders in the soils of the three groups were Rhizobiales, Burkholderiales, and Acidobacteriales, which composed 6.15–9.96%, 5.60–9.57%, and 3.22–9.90% of the total bacteria, respectively. Among them, the dominant order in the SG group appeared to be Gemmatales, which composed 8.71–11.44% of the total bacteria. Rhizobiales and Acidobacteriales had the highest relative abundance in the SD group, and Burkholderiales had the highest relative abundance in the SG group. Overall, the bacterial communities in the soil of the three different farming modes were similar in composition at the phylum and order levels, but there were differences in relative abundance. In addition, there were slight differences in the dominant genera.

A total of 1576 bacterial genera were detected at the genus level, and the samples were clustered using the Bray–Curtis distance. As shown in Figure 2c, the SG_4, SD_2, and SG_3 samples clustered into one branch; the SG_1 and SD_4 samples clustered into one branch; the SD_3 and SD_1 samples clustered into one branch, and all the SF samples clustered into one branch. The results showed that the inputs of *Pelophylax nigromaculatus* in the rice–frog co-cropping modes had a significant effect on the soil bacterial flora, but there was no significant difference in the community composition of the soil bacterial flora among the different input densities.

### 3.3. Alpha-Diversity Analysis of the Soil Bacterial Communities under Three Different Farming Modes

The alpha-diversity indices of the soil bacterial communities in the rice–frog fields of different densities are shown in Table 1. The Good’s coverage of 16S rRNA amplicon sequencing was 100%, which indicates that the sequencing results were consistent with the actual microorganisms in the soil samples. In addition, the length of the sequencing met the requirements of the subsequent analysis. Both the Shannon and Simpson indices reflect the diversity of the bacterial community, and a larger Shannon index value and a smaller Simpson index value indicate that there is a higher diversity of species in the community [45]. As shown in Figure S2, the sparse curves of the Chao1 and Shannon indices of the soil bacteria tended to flatten after the sequencing had reached a certain depth and no longer increased with the increase in sequencing depth. This indicates that the number of sequences was sufficient to reflect the diversity of bacteria in the samples. As shown in Table 1, the Shannon index was more uniform in the different groups, and the overall level of the SG group was higher than that of the SF group. These results indicate that the input of *Pelophylax nigromaculatus* in the paddy fields would increase the diversity of the soil bacterial community.

**Figure 3:**
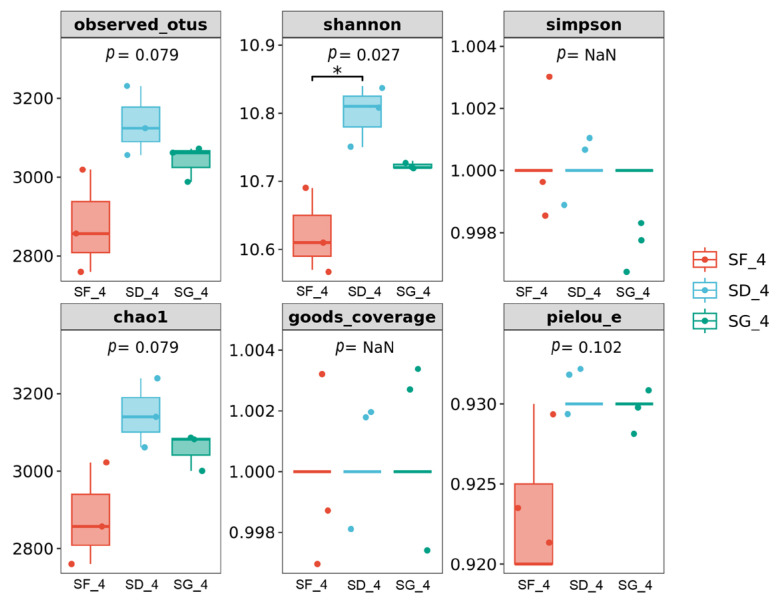
Box plots of the differences in the alpha-diversity indices of soil bacteria in rice–frog fields of different densities. * *p* < 0.05.

The box plots of the alpha-diversity indices for each group in the soil in the rice maturity period showed that although there were no significant differences in the observed OTUs, Simpson, Chao1, Coverage, and Pielou_e indices among the groups, the Shannon index of the SD group was significantly greater than that of the SF group (*p* < 0.05). This suggests that low-density *Pelophylax nigromaculatus* inputs would significantly increase the soil microbial diversity (Figure 3).

### 3.4. Beta-Diversity Analysis of the Soil Bacterial Community Structure under Three Different Farming Modes

**Figure 4:**
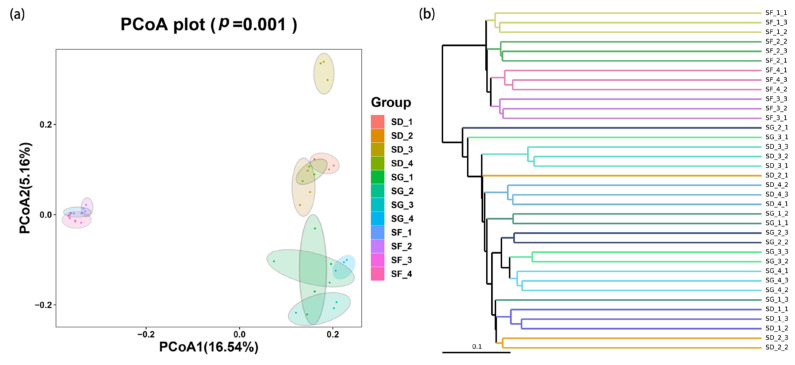
Beta-diversity analysis of the soil bacterial community structures in the rice–frog fields at different densities: (**a**) principal coordinate analysis (PCoA) based on a weighted Bray–Curtis for the soil bacteria; (**b**) phylogenetic tree reconstruction of the soil microorganisms using the unweighted pair group method with arithmetic means (UPGMA).

The PCoA shows the variation in the soil bacterial communities in the rice–frog fields of different densities. The PCoA1 and PCoA2 axes contributed 16.54% and 5.16% of the total bacterial variance, respectively, with a cumulative contribution of 21.7% (Figure 4a). Both the SD and SG groups are on the positive half-axis of the PCoA1, with no significant deviation from the confidence interval ellipse, and the bacterial compositions were relatively similar. In contrast, the SF group was distributed on the negative half-axis of PCoA1 with a significant deviation, which indicates that there were differences in the bacterial compositions of the rice monoculture and rice–frog co-cropping groups. In the comparison of the soil bacteria in the high-density rice–frog group in different periods, the confidence intervals of the SG_1–4 groups overlapped slightly. The same phenomenon also appears in the SD and SF groups, which indicates that the soil bacterial community compositions of the rice–frog field with the same density was similar in different periods.

As shown in the UPGMA clustering tree (Figure 4b), the bacterial community compositions within the SF, SD, and SG groups were similar, while the bacterial community compositions among the groups were somewhat different. The SD and SG groups clustered into a single unit, while the SF group was alone. This indicated that the rice–frog co-cropping altered the structure of the soil microbial communities compared to the conventional rice monoculture mode.

### 3.5. Analysis of Significant Differences among the Groups of Soil Samples

**Figure 5:**
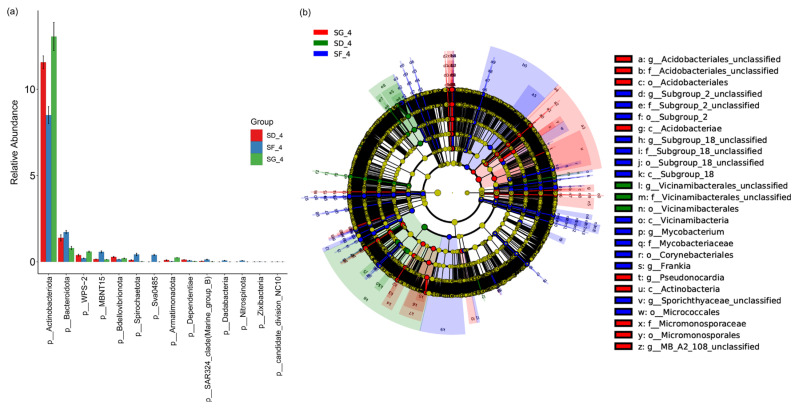
Analysis of significant differences among the groups of soil samples in the rice maturity stage: (**a**) bar plot analysis of the differences among the three groups at the phylum level; (**b**) differential analysis of the LEfSes for species at seven taxonomic levels, from the phylum to genus levels, showing the different abundance taxa of soil microorganisms in rice–frog fields at different densities. More information on the differential taxa is provided in Supplementary Figures S3 and S4. LEfSes, linear discriminant analysis effect sizes.

A bar plot difference analysis was performed at the phylum level to screen for species with a higher abundance at *p* < 0.05 (Figure 5a). The relative abundances of Actinobacteria, WPS-2, and Armatimonadota were the highest in the soil of the SG group, followed by the SD and SF groups, respectively. However, the relative abundances of Bacteroidota, MBNT15, Spirochaetota, and Sva0485 were the highest in the SF group, followed by the SD and SG groups, respectively. Differences in the microbial communities were more pronounced at a deeper taxonomic level, with a total of 175 significantly different species according to the LEfSe analyses at the phylum–genera levels (*p* < 0.05; LDA score ≥ 3). A total of 31 species were enriched in the SG group, 115 species in the SF group, and 29 species in the SD group (Figure 5b and Figure S3).

### 3.6. Predictive Analysis of the Microbial Functions of the Soil Samples from the Rice–Frog Fields at Different Densities

**Figure 6:**
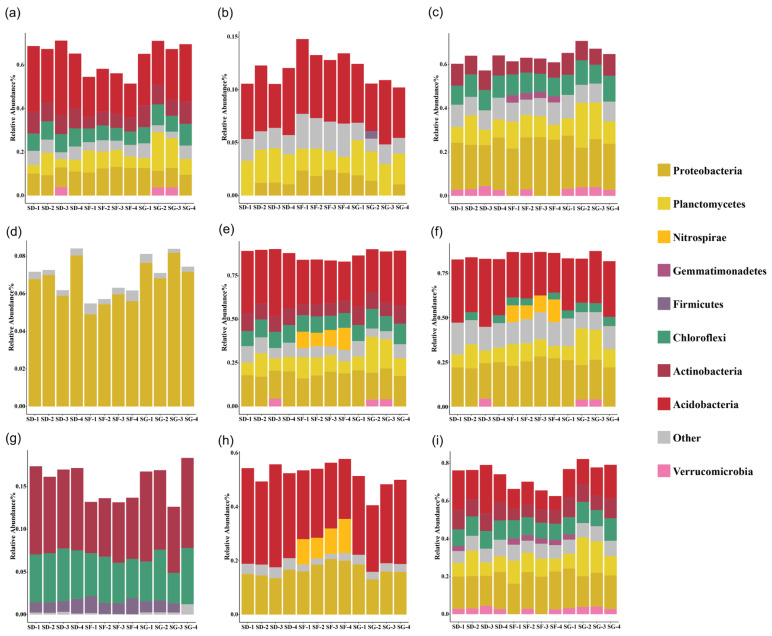
BugBase phenotypic prediction: (**a**) aerobic; (**b**) anaerobic; (**c**) contains mobile elements; (**d**) facultatively anaerobic; (**e**) forms biofilms; (**f**) Gram-negative; (**g**) Gram-positive; (**h**) potentially pathogenic; (**i**) stress tolerant.

The results of the microbial phenotype prediction of the microbiome samples using the BugBase tool are shown in Figure 6. Compared with the SF group, the relative abundances of the aerobic, facultatively anaerobic, stress-tolerant, biofilm-forming, and Gram-positive genes increased in the soils of the SD and SG groups, whereas the relative abundances of the anaerobic, Gram-negative, and potentially pathogenic genes decreased. However, the relative abundance of mobile elements genes in the soils of the SD and SG groups decreased and increased, respectively.

### 3.7. Correlations of the Soil Chemical Properties with the Microbial Communities

**Figure 7:**
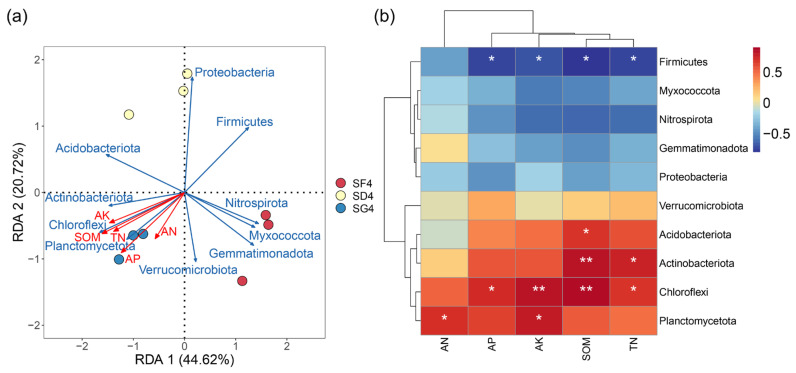
Correlations of the soil chemical properties with the microbial communities: (**a**) redundancy analysis (RDA) of the relationships between the dominant bacteria at the phylum level and soil chemical properties; (**b**) Pearson’s correlation coefficient analysis between the level of bacterial phyla and soil chemical properties. Red and blue indicate positive and negative correlations, respectively. AK: available potassium; AN: available nitrogen; AP: available phosphorus; SOM: soil organic matter; TN: total nitrogen. * *p* < 0.05 and ** *p* < 0.01.

The relationships between the soil chemical properties and microbial community structure were analyzed with an RDA by selecting the top 10 phyla of soil abundance in the mature stage of rice (Figure 7a). The RDA1 and RDA2 contributed a total of 65.34% of the total variance, and the contents of AP, TN, SOM, AK, and AN in the soil were the key factors. The relationships between the soil properties and bacterial community composition were further explored by a Pearson correlation analysis, as shown in Figure 7b. The soil bacterial community of Acidobacteriota was significantly positively correlated with the content of SOM at the phylum level (*p* < 0.05). Actinobacteriota was significantly positively correlated with the content of TN (*p* < 0.05) and highly significant positively correlated with the content of SOM (*p* < 0.01). Chloroflexi was positively correlated with the contents of TN and AP (*p* < 0.05 for both) and highly significant positively correlated with the contents of SOM and AK (*p* < 0.01 for both). Planctomycetota was significantly positively correlated with the contents of AK and AN (*p* < 0.05 for both). However, Firmicutes was significantly negatively correlated (*p* < 0.05) with the contents of AP, TN, SOM, and AK in the soil.

## 4. Discussion

### 4.1. Effects of Different Frog Densities on the Soil Chemical Properties of Reclaimed Paddy Fields

The restoration of reclaimed soil fertility quality is the key to the success of land reclamation, and soil chemical properties were used in this study to reflect the status of fertility in reclaimed fields that had been restored. Rice–frog co-cropping has a certain role in regulating and buffering the soil nutrients in paddy fields [34,46,47], but there has been only limited study on the reclamation of soil transformation using the rice–frog co-cropping mode. At the stage of rice maturity, the content of TN in the soil tended to increase compared with its content before land reclamation, and the content of TN in the soil of the high-density rice–frog co-cropping group (SG) was significantly higher than that of the rice monocropping group (SF). This result is consistent with the findings of Arunrat et al. [36], which indicated that rice–fish co-cropping significantly increased the content of TN of the soil. It was hypothesized that the frog excrement could provide rice with the nitrogen (N) needed for growth and development, thus, meeting the requirement of rice for normal growth. Secondly, the predatory ability of frogs could inhibit a number of pests and thus, reduce the possibility of N loss. Additionally, the feed for the frogs is also rich in nutrients, and this uneaten feed could also permeate the soil and increase its content of N. However, there was no significant difference in the content of AN among the three groups at the stage of rice maturity. This result is inconsistent with the results of some other studies, which indicated that rice–aquatic animal co-culture could increase the content of AN in this soil [31,34,37]. This could be due to frogs excreting nitrogen as urea or ammonia depending on their environment. Nitrogen availability (in nitrate form) might take longer compared to fish, which excrete ammonia. Alternatively, as the initial remediation, the bacterial population may not be robust enough to convert N into AN. The contents of SOM and AK in the soil with the high-density rice–frog co-cropping treatment (SG) were significantly higher than those in the soil with the rice monocropping treatment (SF), and the contents of SOM and soil AK increased with the increased density of the frogs in the paddy field. The results were consistent with those of Yi et al. [34] and Wang et al. [37], who showed that the content of AK increased significantly increased in the rice–frog co-cropping group, and the content of SOM increased in the rice–turtle co-cropping group, respectively, when compared with the rice mono-cropping group. In this study, except for the high-density rice–frog co-cropping group (SG), the soil AP did not significantly increase in the rice maturity period compared with that before reclamation of the field. This is inconsistent with some previous studies in which the rice–aquatic animal co-culture mode significantly increased the content of AP in the soil [36,37,47]. This could be owing to the poor soil fertility and weak biological conditions of the reclaimed fields in this study. There could have been too few frogs in the low-density rice–frog co-cultivation group (SD), which made it more difficult to change the AP content of the soil. Nevertheless, the content of AP in the soil increased significantly in the high-density rice–frog co-cropping group (SG) owing to the higher number of frogs and the supplementation of the AP in the soil by the frog excrement increased the content of AP in the soil. In addition, the various activities of the frogs, such as making holes, turning over the soil, and jumping among others, could improve the aeration of the soil and promote microbial activities and nutrient cycling, thereby increasing the content of AP in the soil.

### 4.2. Effects of Different Frog Densities on the Soil Microorganisms in Reclaimed Paddy Fields

The community structure of microorganisms in soil is an important physiological indicator of soil ecology and plays a crucial role in regulating crop productivity, soil carbon cycling, nutrient transformation, and other important functions [48]. Changes in the soil environment commonly impact the structure of the microbial community in a field [49,50,51,52]. In this study, the soil of the rice monoculture group (SF), low-density rice–frog co-cropping group (SD), and high-density rice–frog co-cropping group (SG) had basically the same composition of bacterial community at the phylum level. The dominant bacterial communities were primarily Proteobacteria, Acidobacteria, Actinobacteria, Planctomycetota, and Chloroflexi. However, the abundance and diversity of the soil bacterial communities in the low-density rice–frog co-cropping group (SD) and the high-density rice–frog co-cropping group (SG) were significantly higher than those in the rice monoculture group (SF). This is because that experimental field is a reclaimed paddy field with low soil fertility, a scattered structure, and a poor ability to retain water and fertilizer, which affects the structure of the soil microbial community. In contrast, the rice–frog co-cropping mode can improve the soil microbial diversity of the paddy field. In addition, with the increase in the density of frogs in the field, the trend of higher contents of bacteria in the soil compared with the rice monocropping group became increasingly apparent.

Proteobacteria and Actinobacteria are eutrophic bacteria, whereas Chloroflexi and Acidobacteria are nutrient-poor bacteria [53,54,55]. Planctomycetota is a key bacterial group that promotes plant growth and plays an important role in nutrient cycling or the control of pathogens [56]. The dominant phyla in the SD group were Proteobacteria, Acidobacteria, and Actinobacteria at different periods in this study. The SG group was enriched in Planctomycetota, and the SF group was enriched in Chloroflexi, probably because of its parthenogenetic anaerobic characteristics. This suggests that the rice–frog co-cropping mode improved the hypoxic environment of the soil. Thus, this study indicates that aeration of the soil was enhanced by the activities of the frogs in the rice–frog co-cropping group. Several studies have shown that Acidobacteria and Chloroflexi grow slowly, as well as in environments that are low in nutrients [57,58]. In this study, the nutrient-poor bacteria in the SF group were significantly higher than those in the SG and SD groups, which was consistent with the results of the soil nutrients, and the enriched Planctomycetota in the SG group was more effective at promoting plant growth and nutrient cycling. These results indicate that the soil nutrient status directly affects the structure of the bacterial community, and rice–frog co-cropping can improve the contents of nutrients in the soil, which, in turn, increases the abundance and diversity of the soil microorganisms.

In this study, the soil Shannon index of the SD group was significantly higher than that of the SF group in the rice maturity stage according to an alpha-diversity analysis (*p* < 0.05), which indicates that low-density rice–frog co-cropping induced changes in the diversity of the soil microbial community and increased the complexity of the soil microbial system. This is consistent with the results of other studies on soil microbial diversity under an integrated rice–frog farming mode [31,36,37]. In contrast, there was no significant difference in the soil alpha-diversity indices between the SD and SF groups, which may be due to the lower density of frogs in the SD group, which was not sufficient to produce a significant difference in the soil alpha-diversity indices.

The results of soil bacterial community PCoA, as well as UPGMA clustering, showed that the bacterial compositions of the SG and SD groups were more similar, while the SF group significantly deviated. This indicates that the bacterial composition of the rice monoculture group differed from that of the rice–frog co-cropping groups. The intra-group community compositions of all three treatment groups were relatively similar, while the bacterial community compositions among the SF, SD, and SG groups exhibited some differences. This difference might be because the various activities of frogs, such as defecation, predation, burrowing, soil turning, and jumping in the paddy field, improved the chemical properties of the soils. Furthermore, the feed for the frogs also contained rich nutrients, which improved the contents of soil nutrients as they infiltrated the soil, thus promoting the growth of certain soil bacteria and, subsequently, influencing the structure of the soil bacterial community.

### 4.3. Soil Chemical Properties and Microbial Correlation in Reclaimed Paddy Fields

Several studies have demonstrated that a soil’s chemical properties can affect the bacterial community composition of a field [31,34,59,60]. In this study, the RDA between the soil chemical properties and soil microorganisms revealed that the contents of TN, AN, AK, SOM, and AP in the soil were the primary environmental factors that affected the distribution of bacterial communities, which is consistent with the results of several previous studies [31,36,37,61]. The results of this study reveal that the contents of N, AK, SOM, and AP are positively correlated with the abundances of Acidobacteriota, Actinobacteriota, Chloroflexi, and Planctomycetota, but negatively correlated with the abundance of Firmicutes. Thus, this study indicates that the factors that regulate soil bacterial community structure in rice–frog co-cropping paddies at different densities are complex and diverse. This was not determined by a single environmental factor but, instead, was the result of the co-regulation of various factors. However, the specific mechanism of the response of soil microorganisms to environmental factors is not yet clear, and further study is merited.

## 5. Conclusions

This study shows that a rice–frog co-cropping model can increase the contents of soil TN, SOM, AK, and AP, thus improving the fertility of reclaimed paddy fields. The predominant bacterial phyla in the soil included Proteobacteria, Acidobacteria, and Actinobacteria, among others. The SG group was enriched with Planctomycetota, a key bacterial group that promotes plant growth, while Chloroflexi, a nutrient-poor bacterial group, was enriched in the SF group. At the stage of rice maturity, the soil Shannon index of the SD group was significantly higher than that of the SF group, which indicates that low-density rice–frog co-cropping induced changes in the diversity of the soil microbial communities and increased the complexity of the soil microbial systems. Significant correlations were found between the levels of TN, AN, AK, SOM, and AP in the soil and the abundance, community diversity, and structure of soil microorganisms, which were the primary environmental factors that influenced the changes in the community of soil microorganisms.

## Figures and Tables

**Table 1:** Alpha-diversity index of the soil bacteria.

Sample ID	Observed Otus	Shannon	Simpson	Chao 1	Coverage	Pielou_e
SF_1	3013 ± 115 ab	10.67 ± 0.06 ab	1	3013 ± 115 ab	1	0.92 ± 0.00 a
SF_2	3076 ± 80 ab	10.71 ± 0.04 ab	1	3077 ± 88 ab	1	0.92 ± 0.01 ab
SF_3	2971 ± 152 ab	10.64 ± 0.10 ab	1	2975 ± 154 ab	1	0.92 ± 0.01 ab
SF_4	2877 ± 129 ab	10.62 ± 0.06 ab	1	2879 ± 132 ab	1	0.92 ± 0.01 ab
SD_1	2998 ± 218 ab	10.64 ± 0.10 ab	1	3006 ± 224 ab	1	0.92 ± 0.00 a
SD_2	3437 ± 463 b	10.91 ± 0.20 b	1	3451 ± 482 b	1	0.93 ± 0.00 c
SD_3	2636 ± 318 a	10.44 ± 0.19 a	1	2648 ± 321 a	1	0.92 ± 0.00 a
SD_4	3135 ± 90 ab	10.80 ± 0.04 b	1	3147 ± 92 ab	1	0.93 ± 0.00 c
SG_1	3055 ± 402 ab	10.77 ± 0.96 ab	1	3066 ± 416 ab	1	0.93 ± 0.00 c
SG_2	3064 ± 912 ab	10.69 ± 0.50 ab	1	3087 ± 925 ab	1	0.93 ± 0.01 bc
SG_3	3141 ± 182 ab	10.79 ± 0.05 ab	1	3160 ± 186 ab	1	0.93 ± 0.00 c
SG_4	3043 ± 44 ab	10.72 ± 0.01 ab	1	3061 ± 40 ab	1	0.93 ± 0.00 c

Values in the same column with different lowercase letters differ significantly from each other (*p* < 0.05). The mean ± SE are shown for the related data.

## Data Availability

Raw sequence data generated for this study are available at the China National GeneBank (CNGB) under BioProject accession number: CNP0005665.

## Supplementary Materials

The following supporting information can be downloaded at: https://www.mdpi.com/article/10.3390/biology13060396/s1, Figure S1: Sparse curves of microbial OTUs in rice–frog fields of different densities; Figure S2. Alpha-diversity analysis of the soil bacterial communities in the rice–frog fields of different densities: (a) Chao1 curve; (b) Shannon exponential curve; Figure S3: Differential analysis of the LEfSes for species from the phylum to genus levels. The cladogram shows the differential abundance taxa (*p* < 0.05, LDA ≥ 3) of the soil microorganisms in rice–frog fields of different densities. The red taxa were significantly enriched in the soil of high-density rice–frog co-cropping fields; the green taxa were significantly enriched in the soil of the low-density rice–frog co-cropping fields, and the purple taxa were significantly enriched in the soil of the rice monocropping fields. LEfSes, linear discriminant analysis effect sizes; Figure S4: Bar plot analysis of the differential abundance taxa with effect size (LDA score). The red taxa were significantly enriched in the soil of the high-density rice–frog co-cropping fields; the green taxa were significantly enriched in the soil of the low-density rice–frog co-cropping fields; and the purple taxa were significantly enriched in the soil of the rice monocropping fields. LDA, linear discriminant analysis.
