# Supplementary material for: Effects of Rice–Frog Co-Cropping on the Soil Microbial Community Structure in Reclaimed Paddy Fields
Source: Biology (Basel). 2024 May 30;13(6):396. doi: 10.3390/biology13060396 (PMC11200385; doi:10.3390/biology13060396)
Supplement: Supplementary file 1 [file biology-13-00396-s001.zip › biology-3024099-supplementary.pdf]

Supplementary Materials

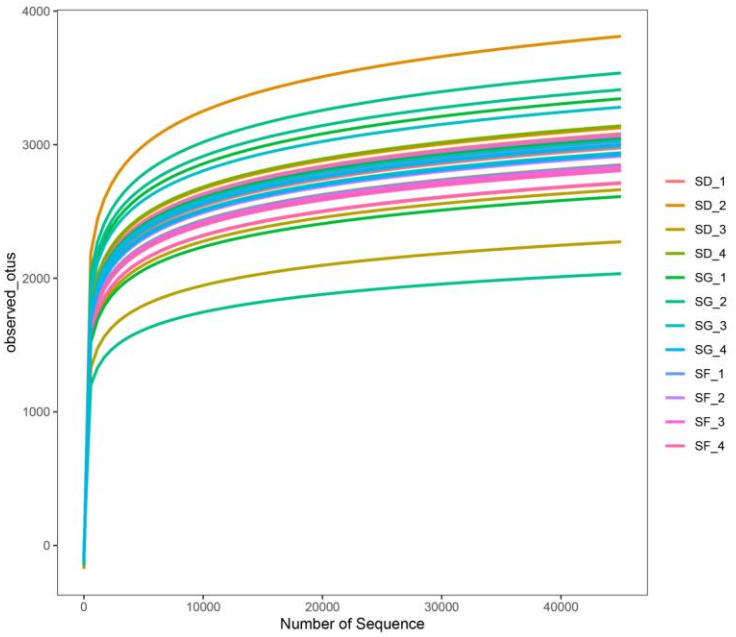

Figure S1. Sparse curves of microbial OTUs in rice–frog fields of different densities.

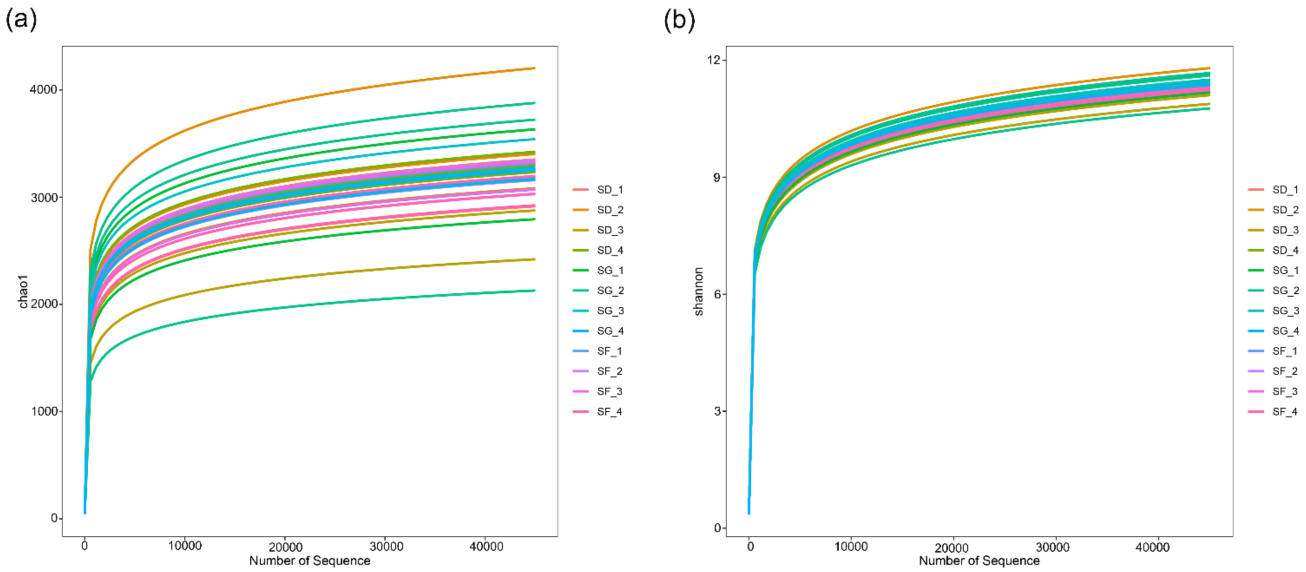

Figure S2. Alpha-diversity analysis of soil bacterial communities in rice-frog fields of different densities: (a) Chao1 curve; (b) Shannon exponential curve.

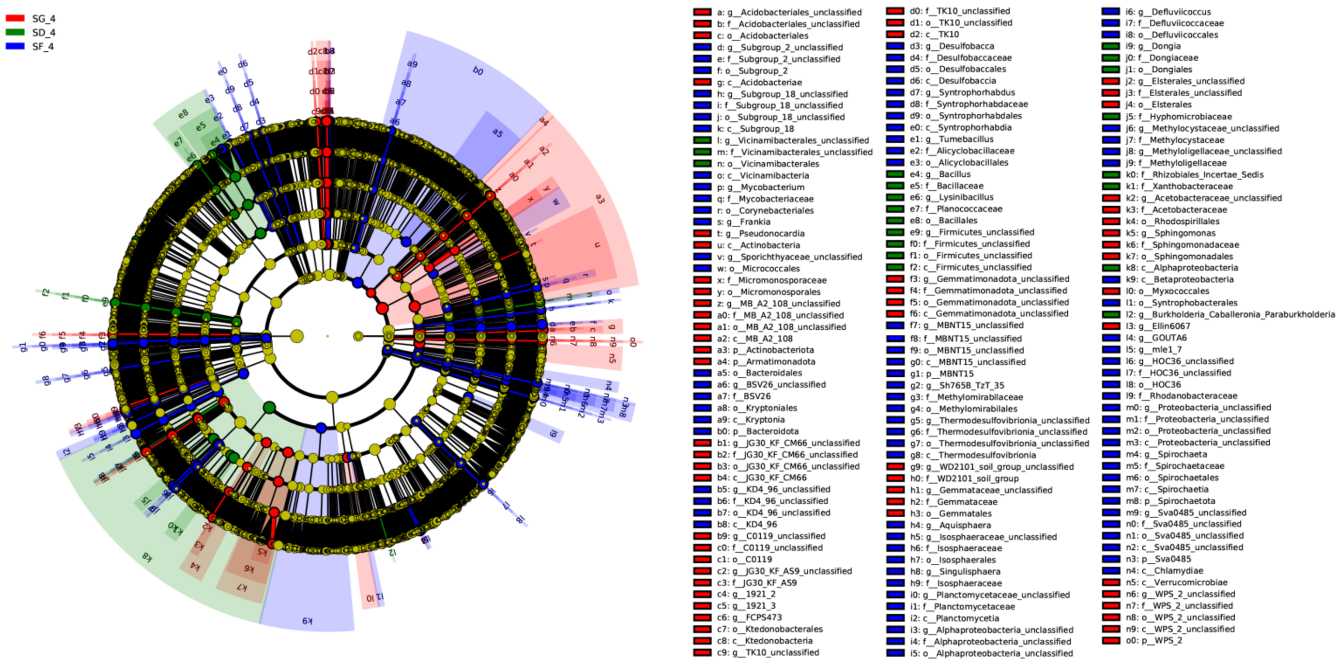

Figure S3. Differential analysis of the LEfSes for species from the phylum to genus levels. The cladogram shows the differential abundance taxa ($p < 0.05$, LDA ≥ 3) of the soil microorganisms in rice–frog fields of different densities. The red taxa were significantly enriched in the soil of the high-density rice–frog co-cropping fields; the green taxa were significantly enriched in the soil of the low-density rice–frog co-cropping fields; and the purple taxa were significantly enriched in the soil of the rice monocropping fields. LEfSes, linear discriminant analysis effect sizes.

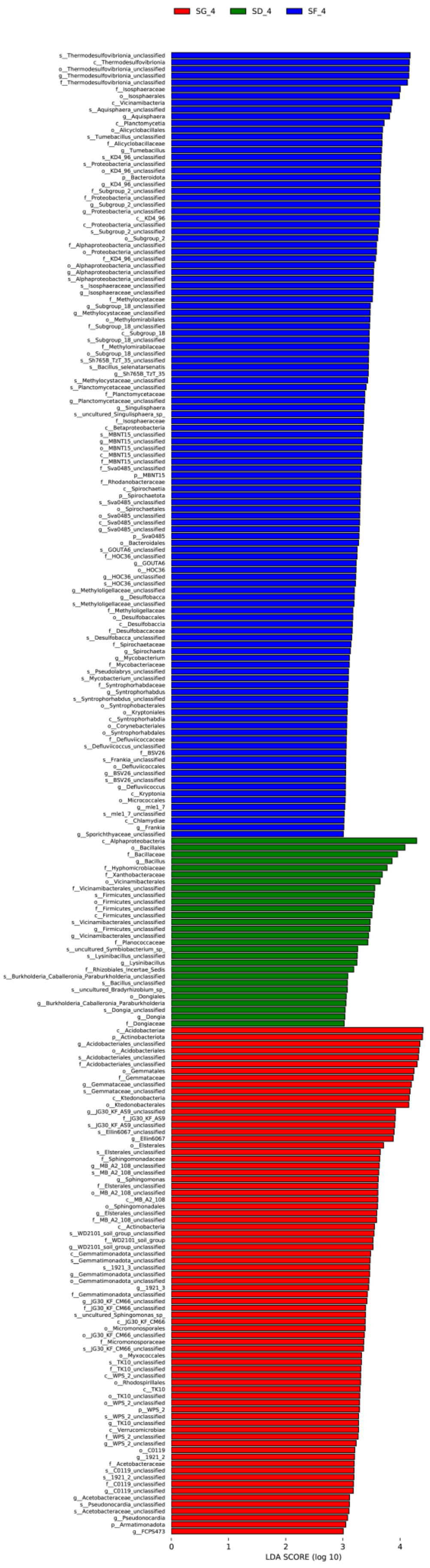

Figure S4. Bar plot analysis of differential abundance taxa with effect size (LDA score). The red taxa were significantly enriched in the soil of high-density rice-frog co-cropping fields; the green taxa were significantly enriched in the soil of low-density rice-frog co-cropping fields, and the purple taxa were significantly enriched in the soil of rice monocropping fields. LDA, linear discriminant analysis.
